# 

**Published:** 1907-08-15

**Authors:** 


CONTENTS

Event and Comment - - - - - - - - - 377
A Discourse on Brains, - - - *By Prof. C. M. Wright* 384
Extracting Abscessed Teeth, - - - *By G. B. Mitchell* 390
Alcohol in Mouth Disinfection, - - *By W. F. Eitch, D.D.S.* 404
Gold Inlays, - - - - *By Charles F. Woodbury, D.D.S.* 408
The Use of Liquid Rubber in Dentistry, *By J. T. Nelson, D.D.S.* 414
Compressed Air, its Uses in Dentistry, *By W. F. Linnert, D.D.S.* 417
Indents - - - - - - - - - - - 420
Announcements - - - - - - - - - - 426

